# Impact of anti-VEGF therapy on choroidal thickness in patients with retinal vein occlusion: a systematic review and meta-analysis

**DOI:** 10.3389/fmed.2025.1663350

**Published:** 2025-12-10

**Authors:** Xiner Cheng, Xinwei Zeng, Shimei Liu, Chang Liu, Yulong Liu, Guoxu Xu

**Affiliations:** 1 Eye Center, The Second Affiliated Hospital, Soochow University, Suzhou, China; 2 Clinical Diagnosis and Treatment Center of Oncology, The Second Affiliated Hospital of Soochow University, Suzhou, China

**Keywords:** retinal vein occlusion, optical coherence tomography, choroid, choroidal thickness, anti-vascular endothelial growth factor, bevacizumab, aflibercept, ranibizumab

## Abstract

**Objectives:**

To systematically review and analyze existing literature reporting changes in choroidal thickness at 1, 3, 6, and 12 months following anti-VEGF treatment in patients with retinal vein occlusion (RVO).

**Methods:**

A comprehensive literature search identified 16 eligible studies including a total of 545 patients and 547 eyes. Data were extracted on subfoveal choroidal thickness at baseline and at specified follow-up intervals. Meta-analyses were performed to assess pooled changes in choroidal thickness after treatment, and subgroup analyses were conducted based on anti-VEGF drug type and treatment regimen.

**Results:**

At 1- and 3-month follow-up, anti-VEGF therapy was associated with a significant reduction in choroidal thickness. By 12 months, choroidal thickness tended to return toward baseline levels, suggesting potential stabilization or structural adaptation. Subgroup analyses indicated that the effects on choroidal thickness may vary among different anti-VEGF agents, with ranibizumab and aflibercept showing significant early reductions, while bevacizumab demonstrated more delayed effects. Further subgroup analyses based on RVO type showed that at 1 month, choroidal thickness was significantly reduced in branch retinal vein occlusion patients, but not in central retinal vein occlusion patients, suggesting potential differences in drug response between RVO subtypes. However, given the limited number of studies within some subgroups, these findings should be interpreted with caution.

**Conclusion:**

In patients with RVO, anti-VEGF therapy appears to induce an early reduction in choroidal thickness, which tends to return toward baseline with longer follow-up. Subgroup analyses provided preliminary insights into the potential influence of different agents, disease subtypes, and treatment regimens. However, this study was limited by the small number of eligible studies and the heterogeneity of study designs. Therefore, the findings should be regarded as exploratory and interpreted with caution. Future prospective studies with larger sample sizes, standardized imaging protocols, and longer follow-up are needed to clarify the clinical relevance of choroidal thickness changes in RVO patients undergoing anti-VEGF therapy.

**Systematic review registration:**

https://www.crd.york.ac.uk/prospero/, identifier CRD 420251011228.

## 1 Introduction

The frequent retinal vascular condition known as retinal vein occlusion (RVO) usually affects middle-aged and older people. Depending on ethnicity, its estimated frequency varies between 0.3% and 2.3% (1). RVO frequently causes retinal blood flow impairment, which can lead to vascular-related blindness and a series of other visual abnormalities. Although the precise etiology of RVO is yet unknown, atherosclerosis, diabetes mellitus, hypertension, and thrombosis are frequently linked to it. RVO risk is also raised by other cardiovascular diseases such heart disease and hyperlipidemia (2). RVO is divided into three categories based on the physical location of vascular blockage: branch retinal vein occlusion (BRVO), superior or inferior retinal vein occlusion, and central retinal vein occlusion (CRVO). The main goals of RVO treatment before the development of anti-VEGF drugs were to control problems, reduce symptoms, and preserve or restore eyesight to the greatest extent feasible. Early management mostly relied on laser therapy or close observation alone because there were no effective etiological treatments, which frequently led to gradual vision deterioration. A new era in RVO treatment began with the advent of anti-VEGF medication, which greatly enhanced patient results and quality of life. Numerous studies comparing the effectiveness of anti-VEGF medications such ranibizumab, bevacizumab, and aflibercept have surfaced as a result of their extensive clinical use. The main outcome measures in these investigations are frequently central foveal retinal thickness and best-corrected visual acuity. Recent studies, however, have demonstrated that the choroid is also involved in the pathogenesis of RVO. Thickness abnormalities in the choroid, a vital circulatory layer supplying the retina, are indicative of the pathophysiological condition in patients with RVO. In RVO patients, choroidal edema is frequently seen, especially in the acute stage. One characteristic of choroidal inflammation and fluid accumulation brought on by RVO is an increase in choroidal thickness. By decreasing neovascularization and vascular permeability, anti-VEGF therapy reduces retinal and choroidal edema; therefore, choroidal thickness is a good measure of the effectiveness of treatment. Previous research has indicated that eyes affected by RVO have higher subfoveal choroidal thickness (SFCT), stromal area, and total choroidal area than eyes in the normal range (3–5). Nevertheless, Alis et al. (6) found no discernible alteration in total choroidal blood flow in patients with RVO, indicating that the rise in SFCT might be due to increased vascular permeability, fluid leakage, and vascular endothelial growth factor (VEGF) levels rather than alterations in vascular density or index. According to some research, SFCT dramatically drops after anti-VEGF therapy, and choroidal thinning might be linked to better treatment results. Other researchers, on the other hand, found no discernible variation in choroidal thickness prior to or following treatment (7, 8). Even though anti-VEGF medication has been the subject of numerous studies, the results are still unclear regarding how it affects choroidal thickness in RVO patients. The purpose of this meta-analysis is to ascertain whether anti-VEGF medication has a systematic impact on choroidal thickness in patients with RVO and whether the effects of various drugs on choroidal thickness vary.

## 2 Methods

### 2.1 Protocol and registration

The study protocol (CRD 420251011228) is registered in PROSPERO. Following the principles set forth by the Preferred Reporting Items for Systematic Reviews and Meta-Analyses (PRISMA), this systematic review and meta-analysis was planned, carried out, and reported (9).

### 2.2 Search strategy

In August 2025, a comprehensive search of the literature was carried out using PubMed, EMBASE, Web of Science, the Cochrane Library, and ClinicalTrials.gov. Search terms included combinations of “retinal vein occlusion, branch retinal vein occlusion, or central retinal vein occlusion,” “vascular endothelial growth factor, anti-VEGF, VEGF inhibitors, or names of specific anti-VEGF agents,” and “choroid or choroidal thickness.” The full search strategy is provided in Supplementary Table 1.

### 2.3 Inclusion and exclusion criteria

Inclusion criteria were as follows: (1) Population: patients with RVO undergoing anti-VEGF therapy for the first time; (2) Intervention: treatment with anti-VEGF agents; (3) Comparison: choroidal thickness before and after anti-VEGF treatment; (4) Outcomes: the primary outcome was the change in choroidal thickness from baseline to different follow-up time points (1, 3, 6, and 12 months). Secondary outcomes included the type of anti-VEGF agent used, RVO subtype, and optical coherence tomography (OCT) device used for measurement; (5) Study types: prospective, retrospective, or cross-sectional cohort studies, case-control studies, or case series were eligible. Only English-language publications were included.

Exclusion criteria were: (1) studies involving combined treatments with agents other than anti-VEGF (e.g., corticosteroids, laser photocoagulation); (2) patients with ocular comorbidities other than RVO, such as diabetic retinopathy; (3) follow-up duration of less than 1 month; (4) sample size less than 10 participants; (5) study types including case reports, reviews, letters, editorials, or conference abstracts; (6) full text unavailable or lack of relevant outcome data.

### 2.4 Study selection and data extraction

Data extraction and literature screening were carried out separately by two reviewers. After using EndNote to eliminate duplicates, the reviewers looked through abstracts and titles to find papers that might be eligible. Complete texts were obtained and evaluated for ultimate incorporation. The two reviewers’ screening results were contrasted. An independent pair of reviewers retrieved data from the included research. Attempts were made to get in touch with the original study authors in cases where data was missing or insufficient. Any differences in screening or extraction were settled by a third reviewer’s decision or discussion. In addition to the aforementioned primary and secondary outcomes, the following data were collected: study title, author, study setting, year of publication, number of patients and affected eyes, age, sex, loss to follow-up or exclusions, OCT device brand, number of injections, RVO duration, inclusion and exclusion criteria, statistical methods used, and ethics approval.

### 2.5 Risk of bias assessment

Using the Joanna Briggs Institute (JBI) Critical Appraisal Checklist for Case Series, two reviewers independently assessed the risk of bias. Each of the ten domains on the checklist could be marked as “Yes,” “No,” “Unclear,” or “Not applicable.” Any disagreements were resolved by consensus or by the judgment of a third reviewer.

### 2.6 Effect measures

Effect measures included changes in choroidal thickness, measured in micrometers (μm) and accompanied by standard deviation (SD), standard error (SE), confidence intervals (CI), or comparable data, from baseline to different post-treatment time points. The vertical distance between the choroid–sclera interface at the foveal center and the outer edge of the retinal pigment epithelium was known as the SFCT (10). In some investigations, SFCT was assessed using caliper tools on enhanced depth imaging optical coherence tomography (EDI-OCT), whereas in others, trained observers averaged several manual measurements.

### 2.7 Synthesis method

Choroidal thickness line graphs were stratified by anti-VEGF agent and plotted at baseline and at all reported time periods (up to 12 months). Only studies that explicitly reported the specific anti-VEGF agent used and employed a single agent throughout were included in these graphs. Excluded studies included medication switching during follow-up or did not provide data by drug type. A single study could contribute many lines or appear in multiple graphs because each line represented a treatment group within the study. In the meta-analysis, only trials that reported mean SFCT and SD or equivalent data at baseline and at 1, 3, 6, and 12 months after therapy were included. After pooling the mean differences, a random-effects model was used to calculate the 95% CIs.

Subgroup analyses based on the type of anti-VEGF drug were conducted. Because choroidal thickness assessed at other locations showed considerable heterogeneity, we only included studies that employed SFCT as the measurement endpoint in order to minimize the risk of bias. We computed pooled values for the meta-analysis using the proper statistical formulae when data were presented independently for distinct subgroups (e.g., CRVO and BRVO). Studies had to report the mean and SD of SFCT at baseline as well as at 1, 3, 6, and 12 months after therapy in order to be included in the meta-analysis. The SD was calculated using the proper statistical techniques in studies that reported only the SE, CI, or descriptive statistics (e.g., median, range). The heterogeneity index (I^2^) statistic was used to evaluate study heterogeneity. Significant heterogeneity was defined as an I^2^ value of more than 50%, which prompted sensitivity tests to investigate possible sources of heterogeneity and evaluate the reliability of the findings. Fixed-effects models were used when I^2^ was less than 50%, while random-effects models were used otherwise. *P*-values less than 0.05 were considered statistically significant differences between pre- and post-treatment data. If the 95% confidence interval for the pooled mean difference included zero, the result was deemed statistically non-significant.

To assess the potential impact of baseline SFCT differences on results, two further analyses were conducted: (1) the baseline SFCT mean and SD were used as covariates in a meta-regression analysis to ascertain whether baseline differences contributed to heterogeneity; (2) the coefficient of variation (CV) of baseline SFCT was computed across studies to assess between-study variability and its contribution to heterogeneity. Egger’s test, Begg’s rank correlation test, and funnel plots created using Stata software (version 17.0) were used to evaluate publication bias. Review Manager (RevMan) software version 5.4.1 was used to conduct meta-analyses, and the findings were displayed as forest plots.

## 3 Results

### 3.1 Study selection

**FIGURE 1:**
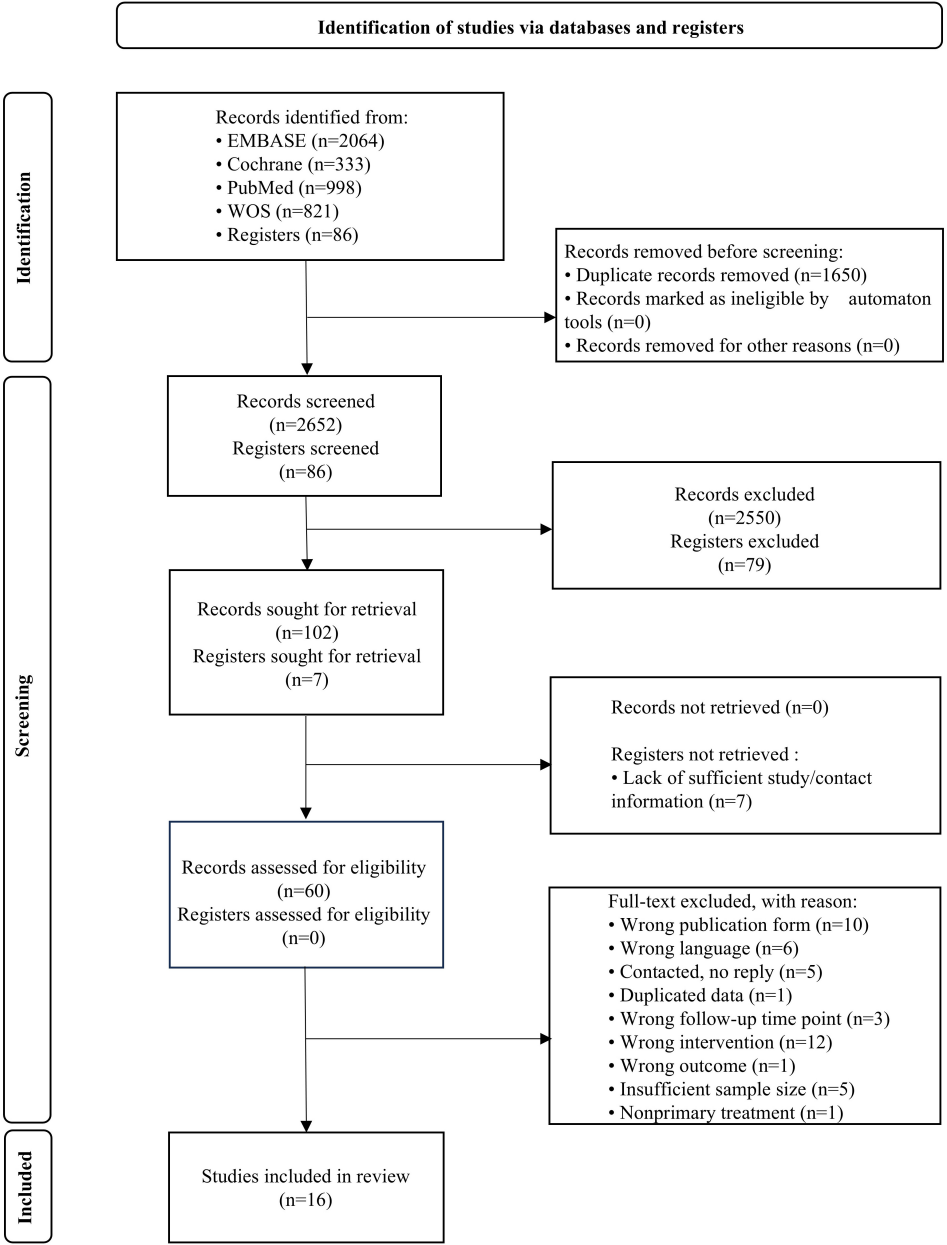
Flow chart of studies identified and included according to the Preferred Reporting Items for Systematic Reviews and Meta-Analyses (PRISMA).

A total of 4,302 records were initially identified through database searches. After removal of duplicates, 2,652 records remained. Titles and abstracts were screened, resulting in the exclusion of 2,550 records that did not meet the predefined inclusion and exclusion criteria. The full texts of 102 articles were then assessed for eligibility. Of these, 42 were excluded after careful evaluation because they did not meet the prespecified inclusion criteria, leaving 60 potentially eligible studies. Among these, further exclusions were applied for specific reasons. Seven register records were not included because no results were submitted. Ultimately, 16 studies satisfied all inclusion requirements (7, 11–21). Figure 1 depicts the comprehensive screening procedure. Four studies did not provide extractable numerical data; instead, they only displayed post-treatment choroidal thickness data graphically or as *p*-values (3, 4, 22, 23). These studies were disqualified because the authors were contacted but did not respond. Three other studies were disqualified because their follow-up intervals (2 or 4 months) did not match (24–26). Four studies that included a small proportion of patients receiving corticosteroids without stratified data were excluded (5, 27–29). One study was excluded because patients who responded poorly to bevacizumab were switched to other anti-VEGF or corticosteroid treatments, and outcomes were not reported separately (30). When data from different studies overlapped, the most recent or thorough publication was chosen. Tracking citations for included studies did not turn up any more relevant information.

### 3.2 Study characteristics

Out of 545 patients, 547 eyes with RVO were included, comprising 303 eyes from 302 patients with BRVO, 215 eyes from 214 patients with CRVO, and 29 eyes from 29 patients with unspecified RVO subtype. Four of the 16 included studies were prospective, and 12 were retrospective. Nine studies reported data at 1 month post-treatment, 10 at 3 months, 5 at 6 months, and 5 at 12 months. Results from fifteen studies were presented as mean ± SD. To prevent data loss, one study’s median, maximum, and minimum results were transformed to mean and SD before being used in the analysis (11). Table 1 provides a summary of the features of the included studies. Only the data from the anti-VEGF treatment arms were extracted for analysis in two studies that compared the effectiveness of corticosteroids and anti-VEGF medications. In a study comparing the efficacy of different doses of bevacizumab, the investigators did not specify the method of dose allocation to patients (19). Since dose effects were not addressed in this meta-analysis, both dose groups were included. The reasoning behind treatment assignment was not stated clearly in the majority of studies. Two studies simply reported the OCT model employed (12, 31), whereas 14 studies used spectral-domain optical coherence tomography (SD-OCT), including EDI-OCT, to quantify choroidal thickness. The most widely used parameter in terms of measuring techniques was SFCT, which was utilized in 14 studies. One study used a seven-point approach to calculate the average choroidal thickness across seven places, including the fovea (11), while two studies measured the central choroidal thickness (12, 31). Some studies performed manual caliper measurements, while others used built-in OCT software. The average value determined by several examiners was reported in the majority of studies.

**TABLE 1:** Baseline characteristics of eligible patients from included studies.

Author	Year	Country	Study design	Number of eyes	Subtype RVO	Anti-VEGF	Measurement type	Treatment protocol
Coban-Karatas	2016	Turkey	Retrospective	42	BRVO	Ranibizumab	Mean of multiple	3 × monthly injections
Dikel	2019	Turkey	Retrospective	17	BRVO	Ranibizumab	SFCT	PRN
Ding	2022	China	Retrospective	35	CRVO, BRVO	Ranibizumab, Aflibercept	CCT	3 + PRN
Dursun	2024	Turkey	Prospective	29	RVO	Bevacizumab	SFCT	3 + PRN
Hashimoto (1)	2024	Japan	Retrospective	16	BRVO	Ranibizumab	SFCT,occlusive region, non-occlusive region	1 + PRN
Hashimoto (2)	2024	Japan	Prospective	20	BRVO	Aflibercept	SFCT, occlusive region, non-occlusive region	1 + PRN
Hwang	2023	South Korea	Retrospective	41	CRVO	Bevacizumab	SFCT	PRN
Kida	2019	Japan	Retrospective	27	CRVO	Aflibercept	CCT	PRN
Kishishita	2022	Japan	Retrospective	36	BRVO	Aflibercept, Ranibizumab	SFCT	PRN
Park	2015	South Korea	Retrospective	41	BRVO	Bevacizumab	SFCT	PRN
Noma	2022	Japan	Prospective	65	BRVO	Ranibizumab	SFCT	Single injection
Rayess	2016	USA	Retrospective	43	CRVO	Bevacizumab, Ranibizumab, Aflibercept	SFCT	3 × monthly injections
Rayess	2018	USA	Retrospective	40	BRVO	Bevacizumab, Ranibizumab	SFCT	3 × monthly injections
Tsuiki	2013	Japan	Retrospective	22	CRVO	Bevacizumab	SFCT	Single injection
Yasuda	2023	Japan	Retrospective	58	CRVO	Ranibizumab	SFCT	Single injection
Yumusak	2016	Turkey	Prospective	15	CRVO, BRVO	Ranibizumab	SFCT, 500 μm NT, 1500 μm NT	PRN

BRVO, branch retinal vein occlusion; CRVO, central retinal vein occlusion; SFCT, subfoveal choroidal thickness; CCT, central choroidal thickness; N, nasal; T, temporal; PRN, pro re nata.

### 3.3 Critical appraisal of studies

To assess the risk of bias, the JBI case series checklist was employed. Loss to follow-up was not reported in all cohort and case series studies, as some were based on databases or medical records, while others were prospective studies. A few studies did not report whether the data’s normality was evaluated, whereas the majority provided adequate statistical reporting. Selection bias may have existed in comparative studies where treatment groups were not assigned at random. However, the risk of bias was deemed minimal for these studies. Supplementary Table 2 presents the risk of bias assessment in detail.

### 3.4 Results of individual studies

**FIGURE 2:**
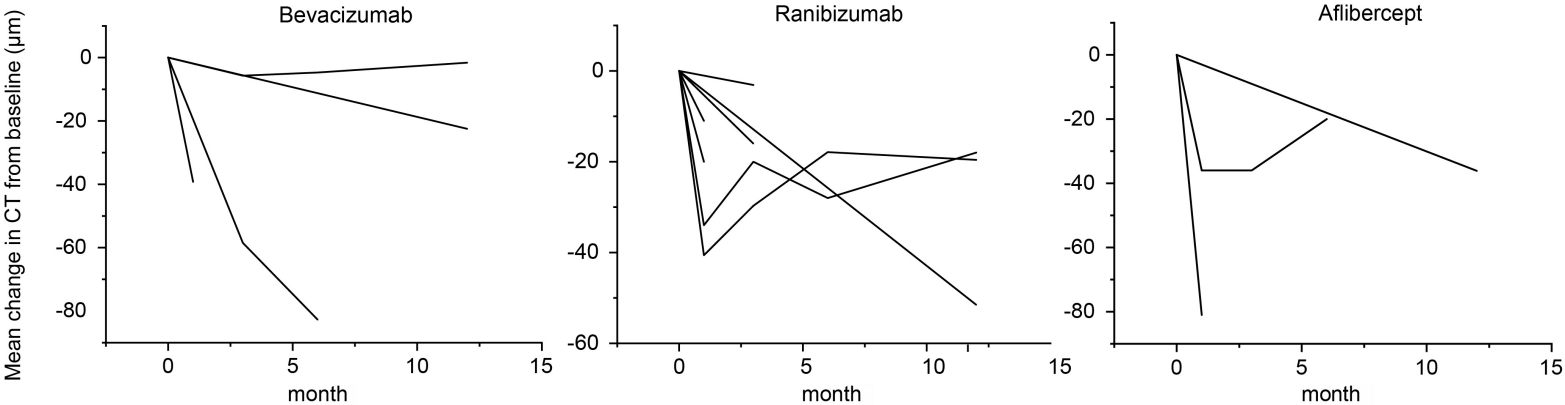
Line chart of changes in choroidal thickness over time between 12 months, according to anti-vascular endothelial growth factor.

Subfoveal choroidal thickness was chosen as the measurement parameter when available; otherwise, central choroidal thickness was used. If a study reported choroidal thickness measurements at multiple locations, we still selected SFCT for analysis. Thirteen studies provided SFCT, two reported central choroidal thickness (within a radius of 1 or 2 mm centered on the fovea) (7, 17), and one study calculated the mean choroidal thickness using a seven-point approach (11). In particular, four studies mentioned that measurements were made during set times because choroidal thickness can change during the day. The follow-up time points (such as 2 or 4 months after therapy) in three trials were not included in this meta-analysis. Changes in choroidal thickness from baseline, stratified by type of anti-VEGF medication, are shown in Figure 2.

### 3.5 Results of synthesis

#### 3.5.1 Meta-analysis of choroidal thickness change

**FIGURE 3:**
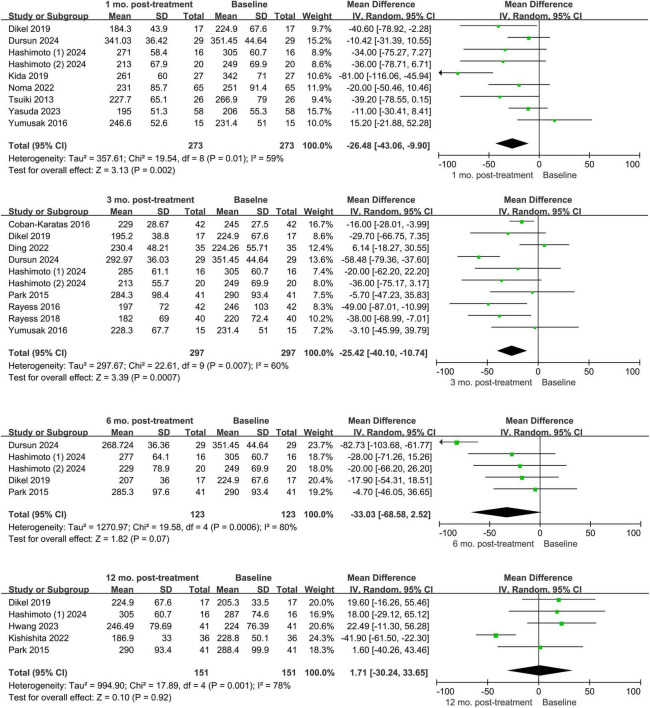
Forest plots of choroidal thickness changes from baseline to different time points for all anti-vascular endothelial growth factor and retinal vein occlusion.

A meta-analysis of 16 trials that reported changes in choroidal thickness from baseline to 1, 3, 6, and 12 months after treatment—including a total of 547 eyes—is shown in Figure 3. Different kinds of anti-VEGF drugs were not distinguished in this investigation. We found that the weighted mean changes in choroidal thickness were -22.17 μm at 1 month (95% CI: −32.17, −12.18), −29.49 μm at 3 months (95% CI: −40.17, −18.81), −49.57 μm at 6 months (95% CI: −64.28, −34.86), and −28.43 μm at 12 months (95% CI: −34.05, −22.80). All follow-up periods showed statistically significant changes in choroidal thickness, with the exception of the 6-month time point (*P* = 0.07). The analysis did not include three studies that reported on various follow-up time points.

#### 3.5.2 Subgroup analysis by type of anti-VEGF agent

**FIGURE 4:**
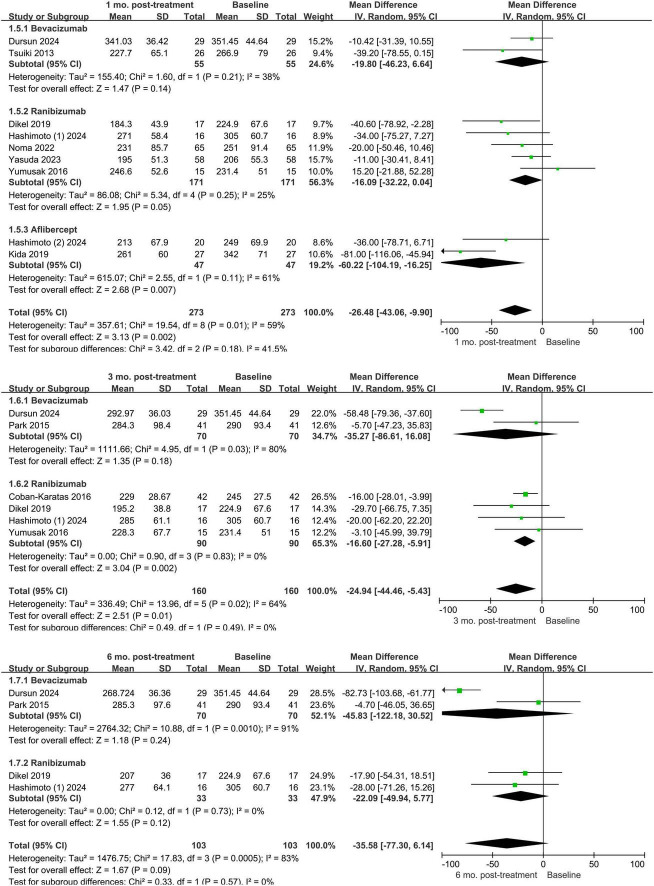
Forest plots of choroidal thickness changes in retinal vein occlusion from baseline to different time points after being divided into different subgroups according to the use of drugs.

Subgroup analyses were carried out based on the kind of anti-VEGF drug used in each study to evaluate changes in choroidal thickness from baseline at various follow-up times, as shown in Figure 4. The included studies involved anti-VEGF agents such as ranibizumab, bevacizumab, and aflibercept. Therefore, subgroup analyses based on the type of anti-VEGF agent were primarily conducted for these three agents. Excluded were four trials that employed many anti-VEGF drugs as treatments (7, 18, 21, 32). We included nine groups for the 1-month follow-up, six groups for 3 months, four groups for 6 months, and five groups for 12 months. At 1 month, the study revealed that while bevacizumab did not significantly reduce choroidal thickness (*P* = 0.08), ranibizumab and aflibercept did (*P* = 0.02 and *P* < 0.00001, respectively). The three subgroups’ differences were statistically significant (*P* = 0.007). While ranibizumab had no discernible effect at 3 months, bevacizumab was linked to a significant decrease in choroidal thickness when compared to baseline (*P* < 0.0001). Between the two subgroups, there was no discernible change (*P* = 0.06). At 6 months, bevacizumab once more showed a significant decrease in choroidal thickness (*P* < 0.00001), although ranibizumab’s effect was still non-significant (*P* = 0.12). A significant difference was found between the subgroups (*P* = 0.0009). At 12 months, neither bevacizumab nor ranibizumab showed statistically significant changes from baseline (*P* = 0.29 and *P* = 0.19, respectively), and no significant differences were observed between subgroups (*P* = 0.81). However, despite utilizing all currently available raw data, the number of studies in the aflibercept and bevacizumab subgroups remains very limited, which may not provide sufficient statistical power for comparisons with the ranibizumab group. Therefore, these findings should be interpreted with caution.

#### 3.5.3 Subgroup analysis based on RVO type

As all included studies categorized RVO solely into BRVO and CRVO, with no separate identification of hemi-CRVO, subgroup analyses were accordingly restricted to these two subtypes. Supplementary Figure 1 shows subgroup analyses that separate research into BRVO and CRVO groups according to the kind of RVO. Due to the limited number of studies, only changes in choroidal thickness from baseline to 1 month after therapy were investigated. Excluded studies were those that did not provide data for BRVO and CRVO separately. At the 1-month follow-up, seven studies were included, three in the CRVO group and four in the BRVO group. According to the findings, choroidal thickness in CRVO patients was not significantly impacted by anti-VEGF medication (*P* = 0.06), but it was dramatically reduced in the BRVO group (*P* = 0.001). This subgroup analysis may reveal potential differences in drug response between different types of RVO; however, the analysis is similarly constrained by the limited data available, and therefore the results should be interpreted only as indicative of possible trends.

#### 3.5.4 Subgroup analysis based on treatment regimen

An additional subgroup analysis based on treatment regimen is shown in Supplementary Figure 2, which divides the studies into pro re nata (PRN) and monthly fixed dosage groups. After being given intravitreally, anti-VEGF drugs are eliminated via the uveoscleral outflow pathway, which may have an impact on the perfusion of choroidal tissue. Therefore, the observed alterations in choroidal thickness in individuals who receive several injections may be partially due to cumulative medication effects inside the choroidal tissue. We conducted a subgroup analysis based on treatment method to take this into consideration. Two kinds of studies were identified: those using a PRN regimen and those using a set monthly injection methodology. The PRN approach usually leads to a smaller cumulative drug exposure than regular monthly doses, which may lessen the degree of drug accumulation and its impact on choroidal structure. Due to the limited number of available studies and the minimal differences in injection frequency between the two subgroups during the early treatment phase (e.g., at 1 month), the present subgroup analysis was restricted to studies reporting outcomes at the 3-month follow-up. The results demonstrated that although both treatment regimens were associated with a significant reduction in choroidal thickness, no statistically significant difference was observed between the two groups. However, since the studies included in the two subgroups showed relatively small differences in the number of injections at the three-month follow-up, this may have introduced bias into the analysis. Therefore, future studies with longer follow-up durations are warranted to further validate these findings.

### 3.6 Heterogeneity and publication bias

In the overall meta-analysis of anti-VEGF treatments, heterogeneity at 1 month was moderate (I^2^ = 59%). To assess the robustness and potential sources of heterogeneity, sensitivity analyses were conducted. After excluding the study by Kida et al. (17), heterogeneity dropped to 10%. At 3 months, heterogeneity was 60% (I^2^ = 60%). After excluding Dursun et al. (13), I^2^ decreased to 29%. At 6 months, high heterogeneity was observed (I^2^ = 80%). Exclusion of Dursun et al. (13) reduced heterogeneity to 0%. At 12 months, heterogeneity was also high (I^2^ = 78%). After excluding Kishishita (33), the heterogeneity was significantly reduced. Forest plots redrawn following sensitivity analysis, with studies that significantly contributed to heterogeneity eliminated, are shown in Supplementary Figure 3. At 1 and 3 months, the results showed a comparatively high level of resilience. Interestingly, when Park (34) was excluded from the 6-month sensitivity analysis, the pooled impact became statistically significant, suggesting that this trial may have affected the final outcome, maybe as a result of variations in medication dosage within the intervention protocol. Although this suggests a degree of robustness in the pooled effect, the findings should still be interpreted with caution. Due to the small number of included studies, we discovered that the 12-month post-treatment results had a comparatively low level of robustness. We then determined that the average baseline choroidal thickness for all studies was 260.86 μm, with a SD of 43.04 μm and a CV of 16.5%. This suggests that there is a moderate amount of variation in baseline choroidal thickness across the studies, which could be a contributing factor to the observed heterogeneity. We also reduced certain sources of heterogeneity by using subgroup analysis based on various anti-VEGF drugs. Heterogeneity could be partially explained by variations in baseline values across studies, according to meta-regression based on baseline choroidal thickness (adjusted R^2^ = 30.70%, *P* = 0.157 at 1 month; R^2^ = 45.37%, *P* = 0.218 at 3 months; R^2^ = 100%, *P* = 0.751 at 6 months; R^2^ = −83.41%, *P* = 0.905 at 12 months). The anti-VEGF dataset’s funnel plots at each follow-up time point are shown in Supplementary Figure 4, which shows comparatively symmetrical distributions. Bias over any period of time, with the exception of the 6-month follow-up. Egger’s test (*P* = 0.2210) and Begg’s test (*P* = 0.3481) showed no publication bias at 1 month after treatment; Egger’s test (*P* = 0.9268) and Begg’s test (*P* = 0.5915) at 3 months; Egger’s test (*P* = 0.0000) and Begg’s test (*P* = 0.8065) at 6 months; and Egger’s test (*P* = 0.0084) and Begg’s test (*P* = 1.0000) at 12 months. The high sensitivity of Egger’s test, which is more vulnerable to the impact of small-sample studies and may exaggerate the existence of bias, could be the cause of the apparent bias seen at 6 months. With the exception of the 6-month follow-up, the data generally hint to no discernible publication bias at any time point.

## 4 Discussion

Within 1 year following treatment for retinal RVO, we systematically reviewed and meta-analyzed studies investigating changes in choroidal thickness at four distinct follow-up time points, with the aim of preliminarily exploring potential trends in choroidal thickness after anti-VEGF therapy and identifying novel indicators that may predict treatment response. The average choroidal thickness considerably decreased from baseline to 1 and 3 months post-treatment in 16 qualifying trials that combined all anti-VEGF drug types and RVO classes. However, at both the 6- and 12-month follow-up visits after treatment, choroidal thickness showed no significant difference compared to baseline. Sensitivity studies were conducted to investigate sources of heterogeneity and validate the robustness of the results. Because Kida’s et al. (17) baseline choroidal thickness was much larger than other studies’, it may have contributed to considerable heterogeneity at 1 month after treatment. Dursun’s (13) study helped to significantly increase heterogeneity at 3 and 6 months after treatment. Although this study’s baseline choroidal thickness was likewise thicker, it was unclear what kind of OCT machine was utilized, and this variation in measurement tools might have added to the studies’ heterogeneity. Heterogeneity at 12 months post-treatment was mostly caused by the Kishishita’s (33) research, which was unique in that it separated BRVO patients into two groups according to whether they were using ranibizumab or aflibercept. The fact that aflibercept was not used in other research may have contributed to the results’ heterogeneity.

By classifying the data according to drug type, we were able to reduce bias in our analysis of choroidal thickness. The type of anti-VEGF medication may help to mitigate the studies’ heterogeneity, according to the results of subgroup analysis. The subgroup analysis in this study indicated that the effects of anti-VEGF agents on choroidal thickness varied significantly across different follow-up time points. Notably, in the two studies conducted by Hashimoto et al. (14), no significant differences in choroidal thickness were observed between ranibizumab and aflibercept at the 1-month post-treatment follow-up. Possible explanations include, on one hand, the small sample sizes of the studies (16 and 20 participants, respectively), which may have introduced considerable bias, and on the other hand, substantial variability in baseline choroidal thickness among patients, which could have influenced the outcomes. Furthermore, the short follow-up duration may have been insufficient to capture potential differences in choroidal thickness between the two agents. However, due to the limited number of studies involving aflibercept and bevacizumab, using these agents as comparators to ranibizumab substantially limits the robustness of the findings. Therefore, the comparison of drug effects in this subgroup analysis should be regarded as preliminary and exploratory. The results demonstrated that ranibizumab significantly reduced choroidal thickness at multiple follow-up time points. Notably, unlike the overall meta-analysis, ranibizumab exhibited a relatively modest effect on choroidal thickness in the early treatment period, with the most pronounced reduction observed at 12 months post-treatment, suggesting that multiple injections may be required for ranibizumab to achieve its maximal therapeutic effect. Furthermore, the inclusion of the trial by Park (34), which employed two different dosages of bevacizumab (1.25 mg/0.05 ml, 2.5 mg/0.05 ml), may have contributed to the fact that heterogeneity in the bevacizumab group increased in this subgroup analysis at both 3 and 6 months. The effect of variations in medication dosage on choroidal thickness following therapy should be investigated in future research. It should be acknowledged that significant heterogeneity across the studies arises from variations in both the types of anti-VEGF agents administered and the treatment regimens employed, thereby imposing substantial limitations on the direct comparability of therapeutic effects.

According to our data, choroidal thickness considerably decreased at the majority of time points following anti-VEGF treatment. Anti-VEGF medications primarily work by preventing neovascularization and decreasing vascular permeability, which may result in less choroidal thickness by lowering choroidal artery leakage and perfusion. Consequently, a decrease in choroidal thickness could indicate a decrease in choroidal perfusion as well as an indirect measure of therapy effectiveness. More research is still needed to determine whether this has long-term effects on RPE function and retinal health. At 1 and 3 months after therapy, we saw a considerable decrease in choroidal thickness, indicating that anti-VEGF medications have a short-term, quick physiological impact on the choroid, with their early effectiveness being more noticeable. The greatest improvement in choroidal thickness occurred 6 months after therapy, however the degree of change did not reach statistical significance, maybe as a result of either a small sample size or significant individual variability leading to some variations. At 12 months post-treatment, choroidal thickness tended to return to baseline levels or even showed slight thickening, suggesting that the initial choroidal thinning may stabilize or partially rebound during the maintenance phase of therapy. This phenomenon may imply the development of “tolerance” or “structural stabilization” with long-term anti-VEGF treatment, or it could reflect diminished therapeutic efficacy due to variations in maintenance regimens. From a pharmacokinetic perspective, anti-VEGF agents are cleared through the uveoscleral outflow pathway, which involves choroidal tissue perfusion. By the 12-month time point, repeated intravitreal injections may lead to localized accumulation of anti-VEGF agents within choroidal structures. This cumulative exposure could potentially induce transient structural alterations in the choroid, such as thickening or changes in vascular permeability. Such pharmacologic accumulation may offer an alternative explanation for the observed increase in choroidal thickness at 12 months.

Therefore, the late-phase thickening of the choroid may reflect either pharmacologic tolerance or drug accumulation effects, and further investigation is warranted to distinguish between these mechanisms. The pattern of choroidal thickness changes is not solely indicative of disease activity or therapeutic response, but may also be influenced by drug clearance kinetics and tissue-level accumulation. Future studies integrating advanced imaging biomarkers with pharmacokinetic modeling are necessary to further elucidate the dynamic interplay between anti-VEGF clearance and choroidal tissue remodeling.

The differences in the effects of various anti-VEGF agents on choroidal thickness may potentially be explained by their pharmacological mechanisms. Despite being anti-VEGF medications, ranibizumab, aflibercept, and bevacizumab have different molecular structures, modes of action, and pharmacokinetic properties. As a fusion protein, aflibercept binds PlGF, VEGF-A, and VEGF-B, perhaps producing a more thorough and long-lasting inhibitory effect. Due to its greater affinity for VEGF-A, ranibizumab shows notable early effects. Even though bevacizumab is a complete antibody, its larger molecular size and lower tissue penetration cause it to act more slowly. In actual practice, these results imply that various anti-VEGF medications might need to be chosen separately depending on the patient’s reaction or the stage of the disease. Aflibercept or ranibizumab may be more appropriate for people who need greater initial treatment responses or who require rapid control of acute edema. Bevacizumab, on the other hand, might provide more affordable and stable structural control for maintenance therapy, which would make it more appropriate for long-term care. The majority of the subgroup studies only contained a few studies (some only two or four studies), and there was variation in dosage, treatment frequency, and baseline patient characteristics despite the differences that the subgroup analysis uncovered. To confirm the long-term effects of anti-VEGF medications on choroidal structure, more prospective research is required, and these data should be viewed cautiously.

In the subgroup analysis based on RVO subtype, a notable difference in treatment response was observed at one month post-injection: patients with BRVO exhibited a significant reduction in choroidal thickness (*P* = 0.001), whereas those with CRVO showed no significant change (*P* = 0.06). This divergence may be attributed to anatomical and hemodynamic differences between the two conditions. In BRVO, the vascular occlusion is localized, typically affecting a single quadrant of the retina, which results in more focal ischemia and a localized inflammatory response. Consequently, anti-VEGF treatment may exert a more direct and measurable impact on choroidal perfusion and structure. In contrast, CRVO represents a more diffuse and extensive venous obstruction, usually involving widespread impairment of retinal and choroidal circulation. This broader damage may attenuate the early structural response of the choroid to anti-VEGF therapy. Additionally, the greater severity and systemic impact of CRVO may require a longer period for detectable morphological changes in the choroid to occur. Therefore, the observed differences in short-term response may reflect the underlying pathophysiological distinctions between these two RVO subtypes.

Regarding the subgroup analysis by treatment regimen, due to the limited number of studies, we were only able to assess choroidal thickness changes at the 3-month follow-up. However, such an analysis may be more informative in studies with longer follow-up periods and repeated injections, as it could better elucidate the cumulative effects of anti-VEGF agents on choroidal structure.

There are various restrictions on this review. The I^2^ did not significantly decrease in several subgroups and in other cases even increased, despite the fact that this study attempted to explain the sources of heterogeneity in the overall analysis by performing subgroup analysis based on the type of anti-VEGF agent the kind of anti-VEGF medicine. Since smaller sample sizes might result in unstable statistical estimations, this phenomenon might be caused by the small number of studies in the subgroups. Additionally, there are still notable variations in treatment frequency, dosage schedules, follow-up periods, and baseline patient characteristics among trials, even within the same drug group, which could potentially induce intra-group heterogeneity. Subgroup studies have limited statistical power and explanatory power because they are exploratory in nature. As a result, the findings should be evaluated cautiously, and future research should improve the analysis of the raw data to identify influencing factors and further standardize intervention techniques. The instruments and techniques used to quantify choroidal thickness also vary from study to study. For choroidal depth imaging, the recommended OCT methods are SS and SD-enhanced depth imaging. Enhanced depth imaging was not specifically reported in a few of the included investigations. Nevertheless, no research was disqualified for this reason. Moreover, because this study only looks at changes in structural indicators and ignores correlations with functional outcomes like visual improvement and the clearance of macular edema, its findings have limited therapeutic utility. Last but not least, a few of the studies in our analysis were small-scale, non-randomized studies without randomization or control. The literature’s overall methodological quality was consequently low. Moreover, due to the limited availability of relevant data, only three anti-VEGF agents were included in the subgroup analysis, and the comparability among these agents was substantially constrained.

## 5 Conclusion

According to this study, anti-VEGF therapy induced a significant reduction in choroidal thickness in the early phase after initiation of treatment in RVO patients, with the effect gradually diminishing over time. This observation highlights the potential trend of choroidal thickness changes following therapy. In the future, based on sufficient evidence, the effects of a broader range of agents on choroidal thickness during long-term treatment should be systematically evaluated.

## Data Availability

The original contributions presented in this study are included in this article/Supplementary material, further inquiries can be directed to the corresponding author.
