# Supplementary material for: Impact of anti-VEGF therapy on choroidal thickness in patients with retinal vein occlusion: a systematic review and meta-analysis
Source: Front Med (Lausanne). 2025 Dec 10;12:1663350. doi: 10.3389/fmed.2025.1663350 (PMC12728066; doi:10.3389/fmed.2025.1663350)
Supplement: Supplementary file 1 [file Table_1.docx]

| **Supplementary Table 1. Literature Search Strategy** | | |
| --- | --- | --- |
| PubMed |  |  |
| Search | Query | Results |
| #1 | "Retinal Vein Occlusion"[Mesh] | 5336 |
| #2 | ((((((((((((((((((((Retinal Vein Occlusion[Title/Abstract]) OR (Occlusion, Retinal Vein[Title/Abstract])) OR (Retinal Vein Occlusions[Title/Abstract])) OR (Vein Occlusion, Retinal[Title/Abstract])) OR (Retinal Vein Thrombosis[Title/Abstract])) OR (Retinal Vein Thromboses[Title/Abstract])) OR (Vein Thrombosis, Retinal[Title/Abstract])) OR (Thrombosis, Retinal Vein[Title/Abstract])) OR (Branch Vein Occlusion[Title/Abstract])) OR (Branch Vein Occlusions[Title/Abstract])) OR (Occlusion, Branch Vein[Title/Abstract])) OR (Vein Occlusion, Branch[Title/Abstract])) OR (Branch Retinal Vein Occlusion[Title/Abstract])) OR (Retinal Branch Vein Occlusion[Title/Abstract])) OR (Central Retinal Vein Occlusion[Title/Abstract])) OR (CRVO[Title/Abstract])) OR (BRVO[Title/Abstract])) OR (retinal vein blockage[Title/Abstract])) OR (retinal vein obstruction[Title/Abstract])) OR (retinal vein thrombosis[Title/Abstract])) OR (RVO[Title/Abstract]) | 7519 |
| #3 | ("Retinal Vein Occlusion"[Mesh]) OR (((((((((((((((((((((Retinal Vein Occlusion[Title/Abstract]) OR (Occlusion, Retinal Vein[Title/Abstract])) OR (Retinal Vein Occlusions[Title/Abstract])) OR (Vein Occlusion, Retinal[Title/Abstract])) OR (Retinal Vein Thrombosis[Title/Abstract])) OR (Retinal Vein Thromboses[Title/Abstract])) OR (Vein Thrombosis, Retinal[Title/Abstract])) OR (Thrombosis, Retinal Vein[Title/Abstract])) OR (Branch Vein Occlusion[Title/Abstract])) OR (Branch Vein Occlusions[Title/Abstract])) OR (Occlusion, Branch Vein[Title/Abstract])) OR (Vein Occlusion, Branch[Title/Abstract])) OR (Branch Retinal Vein Occlusion[Title/Abstract])) OR (Retinal Branch Vein Occlusion[Title/Abstract])) OR (Central Retinal Vein Occlusion[Title/Abstract])) OR (CRVO[Title/Abstract])) OR (BRVO[Title/Abstract])) OR (retinal vein blockage[Title/Abstract])) OR (retinal vein obstruction[Title/Abstract])) OR (retinal vein thrombosis[Title/Abstract])) OR (RVO[Title/Abstract])) | 8480 |
| #4 | (((((("Bevacizumab"[Mesh]) OR "Ranibizumab"[Mesh]) OR "Vascular Endothelial Growth Factors"[Mesh]) OR "aflibercept" [Supplementary Concept]) OR "faricimab" [Supplementary Concept]) OR "brolucizumab" [Supplementary Concept]) OR "KH902 fusion protein" [Supplementary Concept] | 81897 |
| #5 | (((((((((((((((((((((((((((((((((((VEGFs[Title/Abstract]) OR (Vascular Endothelial Growth Factors[Title/Abstract])) OR (VEGF[Title/Abstract])) OR (Bevacizumab[Title/Abstract])) OR (Avasti[Title/Abstract])) OR (Mvasi[Title/Abstract])) OR (Bevacizumab-awwb[Title/Abstract])) OR (Bevacizumab awwb[Title/Abstract])) OR (Ranibizumab[Title/Abstract])) OR (RhuFab V2[Title/Abstract])) OR (V2, RhuFab[Title/Abstract])) OR (Lucentis[Title/Abstract])) OR (Aflibercept[Title/Abstract])) OR (VEGF-Trap[Title/Abstract])) OR (VEGF Trap – regeneron[Title/Abstract])) OR (VEGF Trap-Eye[Title/Abstract])) OR (AVE 005[Title/Abstract])) OR (AVE-005[Title/Abstract])) OR (AVE005[Title/Abstract])) OR (AVE 0005[Title/Abstract])) OR (AVE-0005[Title/Abstract])) OR (AVE0005[Title/Abstract])) OR (Eylea[Title/Abstract])) OR (ZIV-aflibercept[Title/Abstract])) OR (Zaltrap[Title/Abstract])) OR (Pegaptanib[Title/Abstract])) OR (Macugen[Title/Abstract])) OR (pegaptanib sodium[Title/Abstract])) OR (Vabysmo[Title/Abstract])) OR (Brolucizumab[Title/Abstract])) OR (RTH258[Title/Abstract])) OR (RTH-258[Title/Abstract])) OR (ESBA1008[Title/Abstract])) OR (ESBA-1008[Title/Abstract])) OR (KH902 fusion protein[Title/Abstract])) OR (KH902 fusion protein[Title/Abstract]) | 109723 |
| #6 | ((((((((((((((((((((((((((((((((((((VEGFs[Title/Abstract]) OR (Vascular Endothelial Growth Factors[Title/Abstract])) OR (VEGF[Title/Abstract])) OR (Bevacizumab[Title/Abstract])) OR (Avasti[Title/Abstract])) OR (Mvasi[Title/Abstract])) OR (Bevacizumab-awwb[Title/Abstract])) OR (Bevacizumab awwb[Title/Abstract])) OR (Ranibizumab[Title/Abstract])) OR (RhuFab V2[Title/Abstract])) OR (V2, RhuFab[Title/Abstract])) OR (Lucentis[Title/Abstract])) OR (Aflibercept[Title/Abstract])) OR (VEGF-Trap[Title/Abstract])) OR (VEGF Trap – regeneron[Title/Abstract])) OR (VEGF Trap-Eye[Title/Abstract])) OR (AVE 005[Title/Abstract])) OR (AVE-005[Title/Abstract])) OR (AVE005[Title/Abstract])) OR (AVE 0005[Title/Abstract])) OR (AVE-0005[Title/Abstract])) OR (AVE0005[Title/Abstract])) OR (Eylea[Title/Abstract])) OR (ZIV-aflibercept[Title/Abstract])) OR (Zaltrap[Title/Abstract])) OR (Pegaptanib[Title/Abstract])) OR (Macugen[Title/Abstract])) OR (pegaptanib sodium[Title/Abstract])) OR (Vabysmo[Title/Abstract])) OR (Brolucizumab[Title/Abstract])) OR (RTH258[Title/Abstract])) OR (RTH-258[Title/Abstract])) OR (ESBA1008[Title/Abstract])) OR (ESBA-1008[Title/Abstract])) OR (KH902 fusion protein[Title/Abstract])) OR (KH902 fusion protein[Title/Abstract])) OR ((((((("Bevacizumab"[Mesh]) OR "Ranibizumab"[Mesh]) OR "Vascular Endothelial Growth Factors"[Mesh]) OR "aflibercept" [Supplementary Concept]) OR "faricimab" [Supplementary Concept]) OR "brolucizumab" [Supplementary Concept]) OR "KH902 fusion protein" [Supplementary Concept]) | 127845 |
| #7 | (("Tomography, Optical Coherence"[Mesh]) OR "Bruch Membrane"[Mesh]) OR "Choroid"[Mesh] | 66219 |
| #8 | (((((((((((((((((((((((((((((Tomography, Optical Coherence[Title/Abstract]) OR (Coherence Tomography, Optical[Title/Abstract])) OR (Optical Coherence Tomography[Title/Abstract])) OR (OCT Tomography[Title/Abstract])) OR (Tomography, OCT[Title/Abstract])) OR (tomography optical coherence[Title/Abstract])) OR (OCT[Title/Abstract])) OR (Bruch Membrane[Title/Abstract])) OR (Complexus Basalis[Title/Abstract])) OR (Bruch's Membrane[Title/Abstract])) OR (Bruchs Membrane[Title/Abstract])) OR (Lamina Basalis Choroideae[Title/Abstract])) OR (Choroid[Title/Abstract])) OR (Choroids[Title/Abstract])) OR (Sattler Layer[Title/Abstract])) OR (Sattler's Layer[Title/Abstract])) OR (Haller's Layer[Title/Abstract])) OR (Haller Layer[Title/Abstract])) OR (Choriocapillaris[Title/Abstract])) OR (nasal choroid*[Title/Abstract])) OR (temporal choroid*[Title/Abstract])) OR (average choroid*[Title/Abstract])) OR (choroidal thickness[Title/Abstract])) OR (CT[Title/Abstract])) OR (subfoveal choroid*[Title/Abstract])) OR (macular choroid*[Title/Abstract])) OR (choriocapillaris thickness[Title/Abstract])) OR (choriocapillaris complex[Title/Abstract])) OR (perifoveal[Title/Abstract])) OR (parafoveal[Title/Abstract]) | 604356 |
| #9 | ((((((((((((((((((((((((((((((Tomography, Optical Coherence[Title/Abstract]) OR (Coherence Tomography, Optical[Title/Abstract])) OR (Optical Coherence Tomography[Title/Abstract])) OR (OCT Tomography[Title/Abstract])) OR (Tomography, OCT[Title/Abstract])) OR (tomography optical coherence[Title/Abstract])) OR (OCT[Title/Abstract])) OR (Bruch Membrane[Title/Abstract])) OR (Complexus Basalis[Title/Abstract])) OR (Bruch's Membrane[Title/Abstract])) OR (Bruchs Membrane[Title/Abstract])) OR (Lamina Basalis Choroideae[Title/Abstract])) OR (Choroid[Title/Abstract])) OR (Choroids[Title/Abstract])) OR (Sattler Layer[Title/Abstract])) OR (Sattler's Layer[Title/Abstract])) OR (Haller's Layer[Title/Abstract])) OR (Haller Layer[Title/Abstract])) OR (Choriocapillaris[Title/Abstract])) OR (nasal choroid*[Title/Abstract])) OR (temporal choroid*[Title/Abstract])) OR (average choroid*[Title/Abstract])) OR (choroidal thickness[Title/Abstract])) OR (CT[Title/Abstract])) OR (subfoveal choroid*[Title/Abstract])) OR (macular choroid*[Title/Abstract])) OR (choriocapillaris thickness[Title/Abstract])) OR (choriocapillaris complex[Title/Abstract])) OR (perifoveal[Title/Abstract])) OR (parafoveal[Title/Abstract])) OR ((("Tomography, Optical Coherence"[Mesh]) OR "Bruch Membrane"[Mesh]) OR "Choroid"[Mesh]) | 622215 |
| #10 | ((((((((((((((((((((((((((((((((Tomography, Optical Coherence[Title/Abstract]) OR (Coherence Tomography, Optical[Title/Abstract])) OR (Optical Coherence Tomography[Title/Abstract])) OR (OCT Tomography[Title/Abstract])) OR (Tomography, OCT[Title/Abstract])) OR (tomography optical coherence[Title/Abstract])) OR (OCT[Title/Abstract])) OR (Bruch Membrane[Title/Abstract])) OR (Complexus Basalis[Title/Abstract])) OR (Bruch's Membrane[Title/Abstract])) OR (Bruchs Membrane[Title/Abstract])) OR (Lamina Basalis Choroideae[Title/Abstract])) OR (Choroid[Title/Abstract])) OR (Choroids[Title/Abstract])) OR (Sattler Layer[Title/Abstract])) OR (Sattler's Layer[Title/Abstract])) OR (Haller's Layer[Title/Abstract])) OR (Haller Layer[Title/Abstract])) OR (Choriocapillaris[Title/Abstract])) OR (nasal choroid*[Title/Abstract])) OR (temporal choroid*[Title/Abstract])) OR (average choroid*[Title/Abstract])) OR (choroidal thickness[Title/Abstract])) OR (CT[Title/Abstract])) OR (subfoveal choroid*[Title/Abstract])) OR (macular choroid*[Title/Abstract])) OR (choriocapillaris thickness[Title/Abstract])) OR (choriocapillaris complex[Title/Abstract])) OR (perifoveal[Title/Abstract])) OR (parafoveal[Title/Abstract])) OR ((("Tomography, Optical Coherence"[Mesh]) OR "Bruch Membrane"[Mesh]) OR "Choroid"[Mesh])) AND (((((((((((((((((((((((((((((((((((((VEGFs[Title/Abstract]) OR (Vascular Endothelial Growth Factors[Title/Abstract])) OR (VEGF[Title/Abstract])) OR (Bevacizumab[Title/Abstract])) OR (Avasti[Title/Abstract])) OR (Mvasi[Title/Abstract])) OR (Bevacizumab-awwb[Title/Abstract])) OR (Bevacizumab awwb[Title/Abstract])) OR (Ranibizumab[Title/Abstract])) OR (RhuFab V2[Title/Abstract])) OR (V2, RhuFab[Title/Abstract])) OR (Lucentis[Title/Abstract])) OR (Aflibercept[Title/Abstract])) OR (VEGF-Trap[Title/Abstract])) OR (VEGF Trap – regeneron[Title/Abstract])) OR (VEGF Trap-Eye[Title/Abstract])) OR (AVE 005[Title/Abstract])) OR (AVE-005[Title/Abstract])) OR (AVE005[Title/Abstract])) OR (AVE 0005[Title/Abstract])) OR (AVE-0005[Title/Abstract])) OR (AVE0005[Title/Abstract])) OR (Eylea[Title/Abstract])) OR (ZIV-aflibercept[Title/Abstract])) OR (Zaltrap[Title/Abstract])) OR (Pegaptanib[Title/Abstract])) OR (Macugen[Title/Abstract])) OR (pegaptanib sodium[Title/Abstract])) OR (Vabysmo[Title/Abstract])) OR (Brolucizumab[Title/Abstract])) OR (RTH258[Title/Abstract])) OR (RTH-258[Title/Abstract])) OR (ESBA1008[Title/Abstract])) OR (ESBA-1008[Title/Abstract])) OR (KH902 fusion protein[Title/Abstract])) OR (KH902 fusion protein[Title/Abstract])) OR ((((((("Bevacizumab"[Mesh]) OR "Ranibizumab"[Mesh]) OR "Vascular Endothelial Growth Factors"[Mesh]) OR "aflibercept" [Supplementary Concept]) OR "faricimab" [Supplementary Concept]) OR "brolucizumab" [Supplementary Concept]) OR "KH902 fusion protein" [Supplementary Concept]))) AND (("Retinal Vein Occlusion"[Mesh]) OR (((((((((((((((((((((Retinal Vein Occlusion[Title/Abstract]) OR (Occlusion, Retinal Vein[Title/Abstract])) OR (Retinal Vein Occlusions[Title/Abstract])) OR (Vein Occlusion, Retinal[Title/Abstract])) OR (Retinal Vein Thrombosis[Title/Abstract])) OR (Retinal Vein Thromboses[Title/Abstract])) OR (Vein Thrombosis, Retinal[Title/Abstract])) OR (Thrombosis, Retinal Vein[Title/Abstract])) OR (Branch Vein Occlusion[Title/Abstract])) OR (Branch Vein Occlusions[Title/Abstract])) OR (Occlusion, Branch Vein[Title/Abstract])) OR (Vein Occlusion, Branch[Title/Abstract])) OR (Branch Retinal Vein Occlusion[Title/Abstract])) OR (Retinal Branch Vein Occlusion[Title/Abstract])) OR (Central Retinal Vein Occlusion[Title/Abstract])) OR (CRVO[Title/Abstract])) OR (BRVO[Title/Abstract])) OR (retinal vein blockage[Title/Abstract])) OR (retinal vein obstruction[Title/Abstract])) OR (retinal vein thrombosis[Title/Abstract])) OR (RVO[Title/Abstract]))) | 998 |
|  |  |  |
| embase |  |  |
| Search | Query | Results |
| #1 | retina vein occlusion'/exp OR 'retina vein occlusion' | 12747 |
| #2 | 'retinal vein occlusion':ab,ti OR 'occlusion, retinal vein':ab,ti OR 'retinal vein occlusions':ab,ti OR 'vein occlusion, retinal':ab,ti OR 'retinal vein thromboses':ab,ti OR 'vein thrombosis, retinal':ab,ti OR 'thrombosis, retinal vein':ab,ti OR 'branch vein occlusion':ab,ti OR 'branch vein occlusions':ab,ti OR 'occlusion, branch vein':ab,ti OR 'vein occlusion, branch':ab,ti OR 'branch retinal vein occlusion':ab,ti OR 'retinal branch vein occlusion':ab,ti OR 'central retinal vein occlusion':ab,ti OR 'crvo':ab,ti OR 'brvo':ab,ti OR 'retinal vein blockage':ab,ti OR 'retinal vein obstruction':ab,ti OR 'retinal vein thrombosis':ab,ti OR 'rvo':ab,ti | 10522 |
| #3 | #1 OR #2 | 14396 |
| #4 | 'vasculotropin'/exp OR 'vasculotropin' OR 'bevacizumab'/exp OR 'bevacizumab' OR 'ranibizumab'/exp OR 'ranibizumab' OR 'aflibercept'/exp OR 'aflibercept' OR 'pegaptanib'/exp OR 'pegaptanib' OR 'faricimab'/exp OR 'faricimab' OR 'brolucizumab'/exp OR 'brolucizumab' OR 'conbercept'/exp OR 'conbercept' | 300479 |
| #5 | 'vasculotropin':ab,ti OR 'vegfs':ab,ti OR 'vascular endothelial growth factors':ab,ti OR 'vegf':ab,ti OR 'avasti':ab,ti OR 'bevacizumab':ab,ti OR 'mvasi':ab,ti OR 'bevacizumab-awwb':ab,ti OR 'bevacizumab awwb':ab,ti OR 'ranibizumab':ab,ti OR 'rhufab v2':ab,ti OR 'v2, rhufab':ab,ti OR 'lucentis':ab,ti OR 'aflibercept':ab,ti OR 'vegf-trap':ab,ti OR 'vegf trap – regeneron':ab,ti OR 'vegf trap-eye':ab,ti OR 'ave 005':ab,ti OR 'ave-005':ab,ti OR 'ave005':ab,ti OR 'ave 0005':ab,ti OR 'ave-0005':ab,ti OR 'ave0005':ab,ti OR 'eylea':ab,ti OR 'ziv-aflibercept':ab,ti OR 'zaltrap':ab,ti OR 'pegaptanib':ab,ti OR 'macugen':ab,ti OR 'pegaptanib sodium':ab,ti OR 'vabysmo':ab,ti OR 'faricimab':ab,ti OR 'brolucizumab':ab,ti OR 'rth258':ab,ti OR 'rth-258':ab,ti OR 'esba1008':ab,ti OR 'esba-1008':ab,ti OR 'conbercept':ab,ti OR 'kh902 fusion protein':ab,ti | 174136 |
| #6 | #4 OR #5 | 310918 |
| #7 | 'optical coherence tomography'/exp OR 'optical coherence tomography' OR 'bruch membrane'/exp OR 'bruch membrane' OR 'choroid'/exp OR 'choroid' | 171928 |
| #8 | optical coherence tomography':ab,ti OR 'tomography, optical coherence':ab,ti OR 'coherence tomography, optical':ab,ti OR 'oct tomography':ab,ti OR 'tomography, oct':ab,ti OR 'tomography optical coherence':ab,ti OR 'oct':ab,ti OR 'bruch membrane':ab,ti OR 'complexus basalis':ab,ti OR 'complexus basali':ab,ti OR 'bruchs membrane':ab,ti OR 'lamina basalis choroideae':ab,ti OR 'choroid':ab,ti OR 'choroids':ab,ti OR 'sattler layer':ab,ti OR 'haller layer':ab,ti OR 'choriocapillaris':ab,ti OR 'nasal choroid*':ab,ti OR 'temporal choroid*':ab,ti OR 'average choroid*':ab,ti OR 'choroidal thickness':ab,ti OR 'ct':ab,ti OR 'subfoveal choroid*':ab,ti OR 'macular choroid*':ab,ti OR 'choriocapillaris thickness':ab,ti OR 'choriocapillaris complex':ab,ti OR 'perifoveal':ab,ti OR 'parafoveal':ab,ti | 1010696 |
| #9 | #7 OR #8 | 1066719 |
| #10 | #3 AND #6 AND #9 | 2064 |
|  |  |  |
| WOS |  |  |
| Search | Query | Results |
| #1 | ((((((((((((((((((((TS=("Retinal Vein Occlusion")) OR TS=("Occlusion, Retinal Vein")) OR TS=("Retinal Vein Occlusions")) OR TS=("Vein Occlusion, Retinal")) OR TS=("Retinal Vein Thrombosis")) OR TS=("Retinal Vein Thromboses")) OR TS=("Vein Thrombosis, Retinal")) OR TS=("Thrombosis, Retinal Vein")) OR TS=("Branch Vein Occlusion")) OR TS=("Branch Vein Occlusions")) OR TS=("Occlusion, Branch Vein")) OR TS=("Vein Occlusion, Branch")) OR TS=("Branch Retinal Vein Occlusion")) OR TS=("Retinal Branch Vein Occlusion")) OR TS=("Central Retinal Vein Occlusion")) OR TS=("CRVO")) OR TS=("BRVO")) OR TS=("retinal vein blockage")) OR TS=("retinal vein obstruction")) OR TS=("retinal vein thrombosis")) OR TS=("RVO") | 8221 |
| #2 | ((((((((((((((((((((((((((((((((((((TS=("Vascular Endothelial Growth Factors")) OR TS=(VEGFs)) OR TS=(VEGF)) OR TS=(Bevacizumab)) OR TS=(Avasti)) OR TS=(Mvasi)) OR TS=("Bevacizumab-awwb")) OR TS=("Bevacizumab awwb")) OR TS=(Ranibizumab)) OR TS=("RhuFab V2")) OR TS=("V2, RhuFab")) OR TS=(Lucentis)) OR TS=(Aflibercept)) OR TS=("VEGF-Trap")) OR TS=("VEGF Trap – regeneron")) OR TS=("VEGF Trap-Eye")) OR TS=("AVE 005")) OR TS=("AVE-005")) OR TS=("AVE005")) OR TS=("AVE 0005")) OR TS=("AVE-0005")) OR TS=("AVE0005")) OR TS=("Eylea")) OR TS=("ZIV-aflibercept")) OR TS=("Zaltrap")) OR TS=("Pegaptanib")) OR TS=("Macugen")) OR TS=("pegaptanib sodium")) OR TS=("Vabysmo")) OR TS=("Faricimab")) OR TS=(Brolucizumab)) OR TS=(RTH258)) OR TS=("RTH-258")) OR TS=("ESBA1008")) OR TS=("ESBA-1008")) OR TS=("KH902 fusion protein")) OR TS=(conbercept) | 149948 |
| #3 | ((((((((((((((((((((((((((((((TS=("Tomography, Optical Coherence")) OR TS=("Coherence Tomography, Optical")) OR TS=("Optical Coherence Tomography")) OR TS=("OCT Tomography")) OR TS=("Tomography, OCT")) OR TS=("tomography optical coherence")) OR TS=("OCT")) OR TS=("Bruch Membrane")) OR TS=("Complexus Basalis")) OR TS=("Complexus Basali")) OR TS=("Bruch's Membrane")) OR TS=("Bruchs Membrane")) OR TS=("Lamina Basalis Choroideae")) OR TS=("Choroid")) OR TS=("Choroids")) OR TS=("Sattler Layer")) OR TS=("Sattler's Layer")) OR TS=("Haller's Layer")) OR TS=("Haller Layer")) OR TS=("Choriocapillaris")) OR TS=("nasal choroid*")) OR TS=("temporal choroid*")) OR TS=("average choroid*")) OR TS=("choroidal thickness")) OR TS=("CT")) OR TS=("subfoveal choroid*")) OR TS=("macular choroid*")) OR TS=("choriocapillaris thickness")) OR TS=("choriocapillaris complex")) OR TS=(perifoveal)) OR TS=(parafoveal) | 766,385 |
| #4 | #2 AND #3 AND #1 | 821 |
|  |  |  |
| cochrane library |  |  |
| Search | Query | results |
| #1 | MeSH descriptor: [Retinal Vein Occlusion] explode all trees | 468 |
| #2 | (Retinal Vein Occlusion):ti,ab,kw OR (Occlusion, Retinal Vein):ti,ab,kw OR (Retinal Vein Occlusions):ti,ab,kw OR (Vein Occlusion, Retinal):ti,ab,kw OR (Retinal Vein Thrombosis):ti,ab,kw OR (Retinal Vein Thromboses):ti,ab,kw OR (Vein Thrombosis, Retinal):ti,ab,kw OR (Thrombosis, Retinal Vein):ti,ab,kw OR (Branch Vein Occlusion):ti,ab,kw OR (Branch Vein Occlusions):ti,ab,kw OR (Occlusion, Branch Vein):ti,ab,kw OR (Vein Occlusion, Branch):ti,ab,kw OR (Branch Retinal Vein Occlusion):ti,ab,kw OR (Retinal Branch Vein Occlusion):ti,ab,kw OR (Central Retinal Vein Occlusion):ti,ab,kw OR (CRVO):ti,ab,kw OR (BRVO):ti,ab,kw OR (retinal vein blockage):ti,ab,kw OR (retinal vein obstruction):ti,ab,kw OR (retinal vein thrombosis):ti,ab,kw OR (RVO):ti,ab,kw | 1179 |
| #3 | #1 OR #2 | 1179 |
| #4 | MeSH descriptor: [Bevacizumab] explode all trees | 3116 |
| #5 | MeSH descriptor: [Ranibizumab] explode all trees | 1213 |
| #6 | (VEGFs):ti,ab,kw OR (Vascular Endothelial Growth Factors):ti,ab,kw OR (VEGF):ti,ab,kw OR (Bevacizumab):ti,ab,kw OR (Avasti):ti,ab,kw OR (Mvasi):ti,ab,kw OR (Bevacizumab awwb):ti,ab,kw OR (Ranibizumab):ti,ab,kw OR (RhuFab V2):ti,ab,kw OR (V2, RhuFab):ti,ab,kw OR (Lucentis):ti,ab,kw OR (Aflibercept):ti,ab,kw OR (VEGF Trap):ti,ab,kw OR (VEGF Trap regeneron):ti,ab,kw OR (VEGF Trap Eye):ti,ab,kw OR (AVE 005):ti,ab,kw OR (AVE005):ti,ab,kw OR (AVE 0005):ti,ab,kw OR (AVE0005):ti,ab,kw OR (Eylea):ti,ab,kw OR (ZIV-aflibercept):ti,ab,kw OR (Zaltrap):ti,ab,kw OR (Pegaptanib):ti,ab,kw OR (Macugen):ti,ab,kw OR (pegaptanib sodium):ti,ab,kw OR (Vabysmo):ti,ab,kw OR (Faricimab):ti,ab,kw OR (Brolucizumab):ti,ab,kw OR (RTH258):ti,ab,kw OR (RTH 258):ti,ab,kw OR (ESBA1008):ti,ab,kw OR (ESBA 1008):ti,ab,kw OR (KH902 fusion protein):ti,ab,kw OR (conbercept):ti,ab,kw | 1213 |
| #7 | #4 OR #5 OR #6 | 15892 |
| #8 | MeSH descriptor: [Tomography, Optical Coherence] explode all trees | 15892 |
| #9 | MeSH descriptor: [Bruch Membrane] explode all trees | 9 |
| #10 | (Tomography, Optical Coherence):ti,ab,kw OR (Coherence Tomography, Optical):ti,ab,kw OR (Optical Coherence Tomography):ti,ab,kw OR (OCT Tomography):ti,ab,kw OR (Tomography, OCT):ti,ab,kw OR (tomography optical coherence):ti,ab,kw OR (OCT):ti,ab,kw OR (Bruch Membrane):ti,ab,kw OR (complexus Basalis):ti,ab,kw OR (Complexus Basali):ti,ab,kw OR (Bruch's Membrane):ti,ab,kw OR (Bruchs Membrane):ti,ab,kw OR (Lamina Basalis Choroideae):ti,ab,kw OR (Choroids):ti,ab,kw OR (Choroid):ti,ab,kw OR (Sattler's Layer):ti,ab,kw OR (Haller's Layer):ti,ab,kw OR (Haller's Layer):ti,ab,kw OR (Haller Layer):ti,ab,kw OR (Choriocapillaris):ti,ab,kw OR (nasal choroid*):ti,ab,kw OR (temporal choroid*):ti,ab,kw OR (average choroid*):ti,ab,kw OR (choroidal thickness):ti,ab,kw OR (CT):ti,ab,kw OR (subfoveal choroid*):ti,ab,kw OR (macular choroid*):ti,ab,kw OR (choriocapillaris thickness):ti,ab,kw OR (choriocapillaris complex):ti,ab,kw OR (perifoveal):ti,ab,kw OR (parafoveal):ti,ab,kw | 94623 |
| #11 | #8 OR #9 OR #10 | 94623 |
| #12 | #3 AND #7 AND #11 | 333 |
