# Supplementary material for: Impact of anti-VEGF therapy on choroidal thickness in patients with retinal vein occlusion: a systematic review and meta-analysis
Source: Front Med (Lausanne). 2025 Dec 10;12:1663350. doi: 10.3389/fmed.2025.1663350 (PMC12728066; doi:10.3389/fmed.2025.1663350)
Supplement: Supplementary file 2 [file Table_2.docx]

| Study | (1) | (2) | (3) | (4) | (5) | (6) | (7) | (8) | (9) | (10) |
| --- | --- | --- | --- | --- | --- | --- | --- | --- | --- | --- |
| Coban-Karatas 2016 | Y | Y | Y | Y | U | Y | Y | Y | Y | Y |
| Dikel 2019 | Y | Y | Y | U | U | Y | Y | Y | Y | Y |
| Ding 2022 | Y | Y | Y | U | U | Y | Y | Y | Y | Y |
| Dursun 2024 | Y | Y | Y | Y | U | Y | Y | Y | Y | Y |
| Hashimoto 2024 (1) | Y | Y | Y | Y | Y | Y | Y | Y | Y | Y |
| Hashimoto 2024 (2) | Y | Y | Y | Y | Y | Y | Y | Y | Y | Y |
| Hwang 2023 | Y | Y | Y | Y | U | Y | Y | Y | Y | N |
| Kida 2019 | Y | Y | Y | Y | U | Y | Y | Y | Y | N |
| Kishishita 2022 | N | Y | Y | U | Y | Y | Y | Y | Y | Y |
| Park 2015 | Y | Y | Y | Y | Y | Y | Y | Y | Y | Y |
| Noma 2022 | Y | Y | Y | U | U | Y | Y | Y | U | Y |
| Rayess 2016 | Y | Y | Y | Y | U | Y | Y | Y | Y | Y |
| Rayess 2018 | Y | Y | Y | Y | U | Y | Y | Y | Y | Y |
| Tsuiki 2013 | N | Y | Y | Y | U | Y | Y | Y | Y | Y |
| Yasuda 2023 | Y | Y | Y | Y | U | Y | Y | Y | Y | N |
| Yumusak 2016 | Y | Y | Y | U | U | Y | Y | Y | Y | N |

**Supplementary Table 2.** JBI critical appraisal quality assessment of the studies.

1. : Clear Criteria for inclusion/exclusion
2. : Reliable measuring
3. : Valid identification methods
4. : Consecutive Inclusion
5. : Complete follow-up
6. : Clear Demographics
7. : Clear Clinical Information
8. : Clear Outcomes
9. : Clear clinic information
10. : Statistical analysis

Y: YES N: NO U: UNCLEAR
