# Supplementary material for: Impact of anti-VEGF therapy on choroidal thickness in patients with retinal vein occlusion: a systematic review and meta-analysis
Source: Front Med (Lausanne). 2025 Dec 10;12:1663350. doi: 10.3389/fmed.2025.1663350 (PMC12728066; doi:10.3389/fmed.2025.1663350)
Supplement: Supplementary file 3 [file Image_1.pdf]

Study or Subgroup	1 mo. post-treatment Mean	SD	Total	Baseline Mean	SD	Total	Weight	Mean Difference IV, Random, 95% CI
**2.1.1 CRVO**								
Kida 2019	261	60	27	342	71	27	14.1%	-81.00 [-116.06, -45.94]
Tsuiki 2013	227.7	65.1	26	266.9	79	26	12.5%	-39.20 [-78.55, 0.15]
Yasuda 2023	195	51.3	58	206	55.3	58	21.5%	-11.00 [-30.41, 8.41]
**Subtotal (95% CI)**			**111**			**111**	**48.0%**	**-42.04 [-85.58, 1.50]**
Heterogeneity: Tau² = 1218.35; Chi² = 12.01, df = 2 (P = 0.002); I² = 83%								
Test for overall effect: Z = 1.89 (P = 0.06)								
**2.1.2 BRVO**								
Dikel 2019	184.3	43.9	17	224.9	67.6	17	12.8%	-40.60 [-78.92, -2.28]
Hashimoto (1) 2024	271	58.4	16	305	60.7	16	11.8%	-34.00 [-75.27, 7.27]
Hashimoto (2) 2024	213	67.9	20	249	69.9	20	11.3%	-36.00 [-78.71, 6.71]
Noma 2022	231	85.7	65	251	91.4	65	16.0%	-20.00 [-50.46, 10.46]
**Subtotal (95% CI)**			**118**			**118**	**52.0%**	**-30.72 [-49.31, -12.13]**
Heterogeneity: Tau² = 0.00; Chi² = 0.81, df = 3 (P = 0.85); I² = 0%								
Test for overall effect: Z = 3.24 (P = 0.001)								
**Total (95% CI)**			**229**			**229**	**100.0%**	**-35.14 [-53.77, -16.52]**
Heterogeneity: Tau² = 322.16; Chi² = 12.84, df = 6 (P = 0.05); I² = 53%								
Test for overall effect: Z = 3.70 (P = 0.0002)								
Test for subgroup differences: Chi² = 0.22, df = 1 (P = 0.64), I² = 0%								

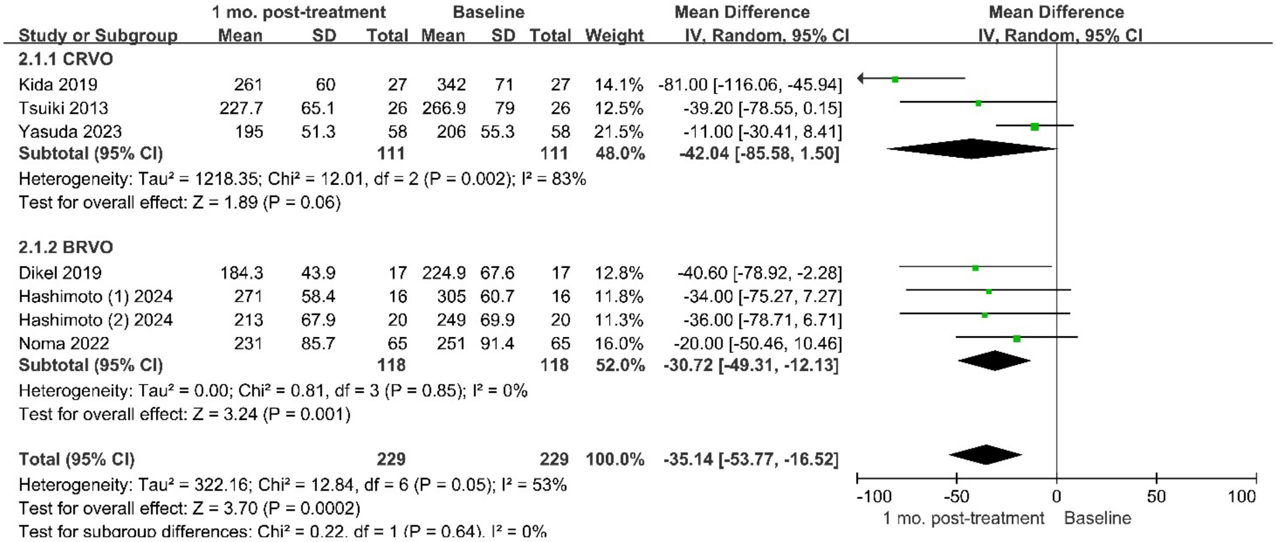

Supplementary Figure 1. Forest plots of choroidal thickness changes from baseline to one month after treatment after being divided into different subgroups according to RVO types
