# Supplementary material for: Impact of anti-VEGF therapy on choroidal thickness in patients with retinal vein occlusion: a systematic review and meta-analysis
Source: Front Med (Lausanne). 2025 Dec 10;12:1663350. doi: 10.3389/fmed.2025.1663350 (PMC12728066; doi:10.3389/fmed.2025.1663350)
Supplement: Supplementary file 4 [file Image_2.pdf]

Study or Subgroup	3 mo. post-treatment Mean	SD	Total	Baseline Mean	SD	Total	Weight	Mean Difference IV, Random, 95% CI
**4.1.1 Monthly fixed**								
Coban-Karatas 2016	229	28.67	42	245	27.5	42	16.7%	-16.00 [-28.01, -3.99]
Ding 2022	230.4	48.21	35	224.26	55.71	35	12.4%	6.14 [-18.27, 30.55]
Dursun 2024	292.97	36.03	29	351.45	44.64	29	13.6%	-58.48 [-79.36, -37.60]
Rayess 2016	197	72	42	246	103	42	8.3%	-49.00 [-87.01, -10.99]
Rayess 2018	182	69	40	220	72.4	40	10.2%	-38.00 [-68.99, -7.01]
**Subtotal (95% CI)**			**188**			**188**	**61.3%**	**-29.73 [-52.92, -6.55]**
Heterogeneity: Tau² = 531.38; Chi² = 20.48, df = 4 (P = 0.0004); I² = 80%								
Test for overall effect: Z = 2.51 (P = 0.01)								
**4.1.2 PRN**								
Dikel 2019	195.2	38.8	17	224.9	67.6	17	8.6%	-29.70 [-66.75, 7.35]
Hashimoto (1) 2024	285	61.1	16	305	60.7	16	7.4%	-20.00 [-62.20, 22.20]
Hashimoto (2) 2024	213	55.7	20	249	69.9	20	8.0%	-36.00 [-75.17, 3.17]
Park 2015	284.3	98.4	41	290	93.4	41	7.5%	-5.70 [-47.23, 35.83]
Yumusak 2016	228.3	67.7	15	231.4	51	15	7.2%	-3.10 [-45.99, 39.79]
**Subtotal (95% CI)**			**109**			**109**	**38.7%**	**-20.01 [-38.07, -1.94]**
Heterogeneity: Tau² = 0.00; Chi² = 1.96, df = 4 (P = 0.74); I² = 0%								
Test for overall effect: Z = 2.17 (P = 0.03)								
**Total (95% CI)**			**297**			**297**	**100.0%**	**-25.42 [-40.10, -10.74]**
Heterogeneity: Tau² = 297.67; Chi² = 22.61, df = 9 (P = 0.007); I² = 60%								
Test for overall effect: Z = 3.39 (P = 0.0007)								
Test for subgroup differences: Chi² = 0.42, df = 1 (P = 0.52), I² = 0%								

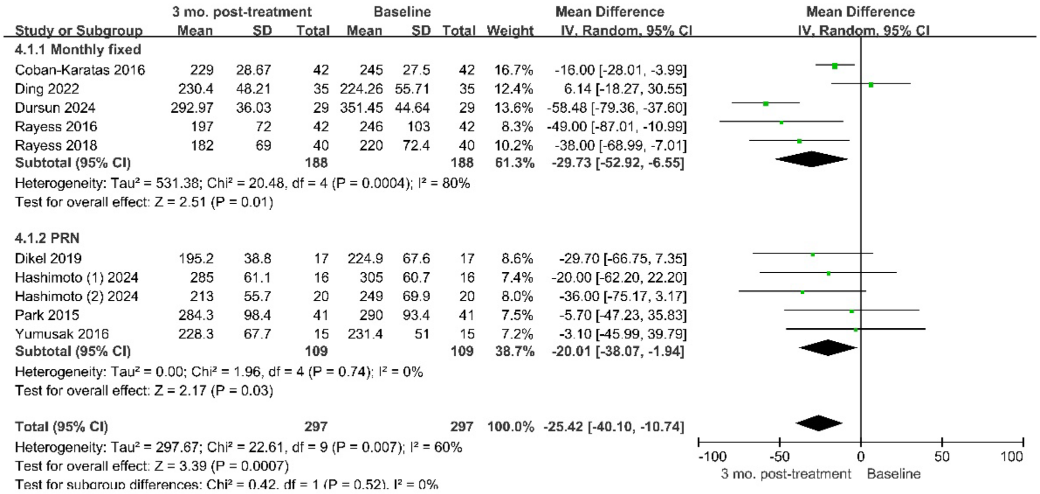

Supplementary Figure 2. Forest plots of choroidal thickness changes from baseline to three months after treatment after being divided into different subgroups according to treatment regimen.
