# Supplementary material for: Impact of anti-VEGF therapy on choroidal thickness in patients with retinal vein occlusion: a systematic review and meta-analysis
Source: Front Med (Lausanne). 2025 Dec 10;12:1663350. doi: 10.3389/fmed.2025.1663350 (PMC12728066; doi:10.3389/fmed.2025.1663350)
Supplement: Supplementary file 5 [file Image_3.pdf]

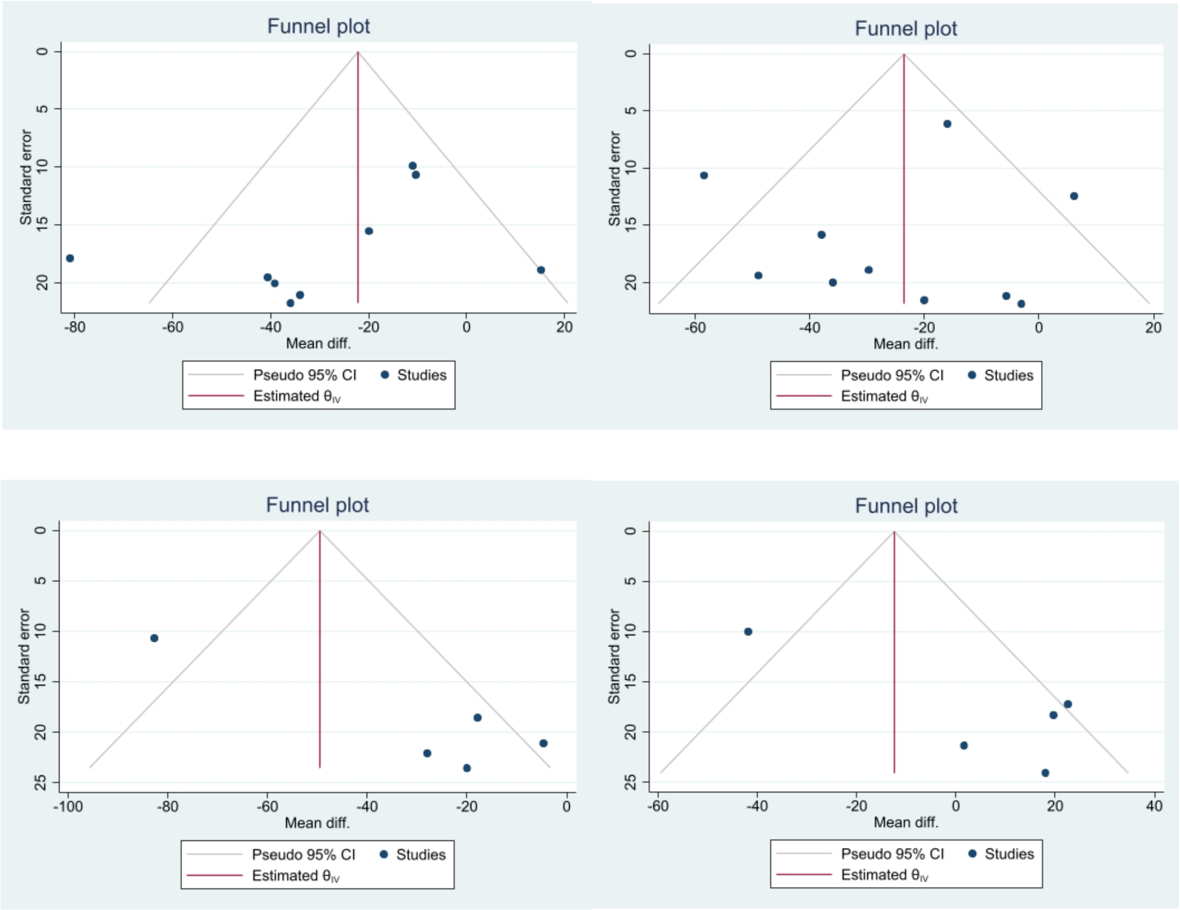

Supplementary Figure 3. The publication bias funnel plots of the included literature at 1 month, 3 months, 6 months and 12 months after treatment.
