# Supplementary material for: Impact of anti-VEGF therapy on choroidal thickness in patients with retinal vein occlusion: a systematic review and meta-analysis
Source: Front Med (Lausanne). 2025 Dec 10;12:1663350. doi: 10.3389/fmed.2025.1663350 (PMC12728066; doi:10.3389/fmed.2025.1663350)
Supplement: Supplementary file 6 [file Image_4.pdf]

	1 mo. post-treatment			Baseline				Mean Difference
Study or Subgroup	Mean	SD	Total	Mean	SD	Total	Weight	IV, Fixed, 95% CI
Dikel 2019	184.3	43.9	17	224.9	67.6	17	7.4%	-40.60 [-78.92, -2.28]
Dursun 2024	341.03	36.42	29	351.45	44.64	29	24.7%	-10.42 [-31.39, 10.55]
Hashimoto (1) 2024	271	58.4	16	305	60.7	16	6.4%	-34.00 [-75.27, 7.27]
Hashimoto (2) 2024	213	67.9	20	249	69.9	20	6.0%	-36.00 [-78.71, 6.71]
Noma 2022	231	85.7	65	251	91.4	65	11.7%	-20.00 [-50.46, 10.46]
Tsuiki 2013	227.7	65.1	26	266.9	79	26	7.0%	-39.20 [-78.55, 0.15]
Yasuda 2023	195	51.3	58	206	55.3	58	28.9%	-11.00 [-30.41, 8.41]
Yumusak 2016	246.6	52.6	15	231.4	51	15	7.9%	15.20 [-21.88, 52.28]
**Total (95% CI)**			**246**			**246**	**100.0%**	**-16.97 [-27.40, -6.54]**

Heterogeneity: Chi² = 7.77, df = 7 (P = 0.35); I² = 10%
Test for overall effect: Z = 3.19 (P = 0.001)

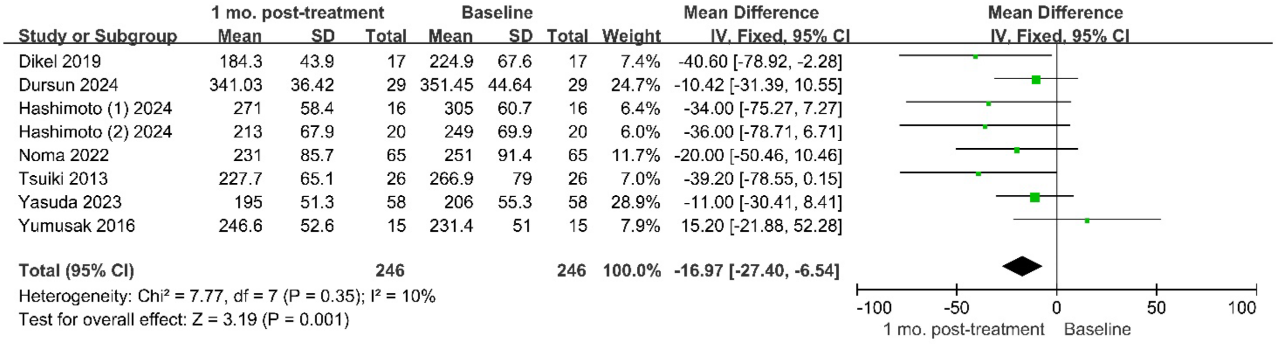

	3 mo. post-treatment			Baseline				Mean Difference
Study or Subgroup	Mean	SD	Total	Mean	SD	Total	Weight	IV, Fixed, 95% CI
Coban-Karatas 2016	229	28.67	42	245	27.5	42	51.7%	-16.00 [-28.01, -3.99]
Dikel 2019	195.2	38.8	17	224.9	67.6	17	5.4%	-29.70 [-66.75, 7.35]
Ding 2022	230.4	48.21	35	224.26	55.71	35	12.5%	6.14 [-18.27, 30.55]
Hashimoto (1) 2024	285	61.1	16	305	60.7	16	4.2%	-20.00 [-62.20, 22.20]
Hashimoto (2) 2024	213	55.7	20	249	69.9	20	4.9%	-36.00 [-75.17, 3.17]
Park 2015	284.3	98.4	41	290	93.4	41	4.3%	-5.70 [-47.23, 35.83]
Rayess 2016	197	72	42	246	103	42	5.2%	-49.00 [-87.01, -10.99]
Rayess 2018	182	69	40	220	72.4	40	7.8%	-38.00 [-68.99, -7.01]
Yumusak 2016	228.3	67.7	15	231.4	51	15	4.1%	-3.10 [-45.99, 39.79]
**Total (95% CI)**			**268**			**268**	**100.0%**	**-17.56 [-26.19, -8.92]**

Heterogeneity: Chi² = 10.01, df = 8 (P = 0.26); I² = 20%
Test for overall effect: Z = 3.98 (P < 0.0001)

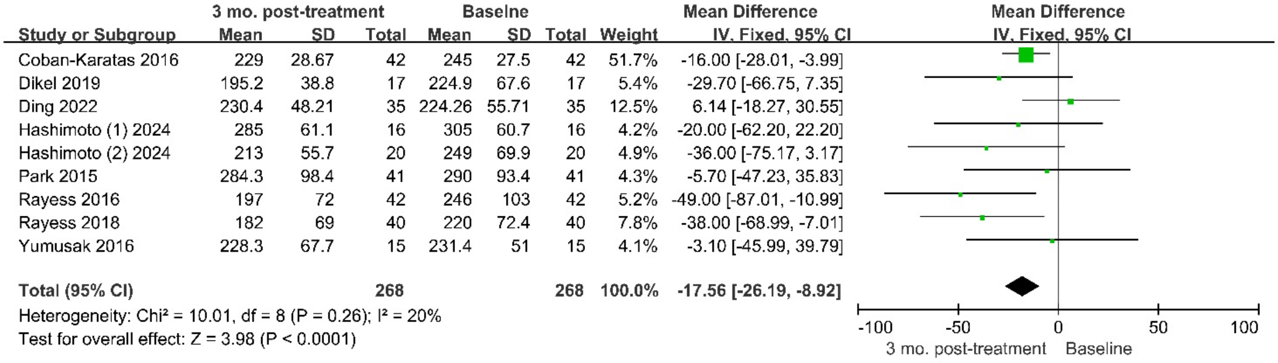

	6 mo. post-treatment			Baseline				Mean Difference
Study or Subgroup	Mean	SD	Total	Mean	SD	Total	Weight	IV, Fixed, 95% CI
Hashimoto (1) 2024	277	64.1	16	305	60.7	16	22.8%	-28.00 [-71.26, 15.26]
Hashimoto (2) 2024	229	78.9	20	249	69.9	20	20.0%	-20.00 [-66.20, 26.20]
Dikel 2019	207	36	17	224.9	67.6	17	32.2%	-17.90 [-54.31, 18.51]
Park 2015	285.3	97.6	41	290	93.4	41	25.0%	-4.70 [-46.05, 36.65]
**Total (95% CI)**			**94**			**94**	**100.0%**	**-17.33 [-37.99, 3.33]**

Heterogeneity: Chi² = 0.61, df = 3 (P = 0.90); I² = 0%
Test for overall effect: Z = 1.64 (P = 0.10)

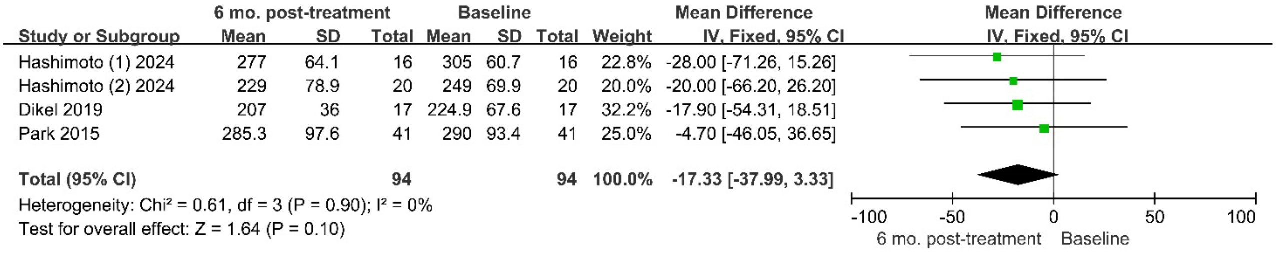

	12 mo. post-treatment			Baseline				Mean Difference
Study or Subgroup	Mean	SD	Total	Mean	SD	Total	Weight	IV, Fixed, 95% CI
Dikel 2019	224.9	67.6	17	205.3	33.5	17	29.1%	19.60 [-16.26, 55.46]
Hashimoto (1) 2024	305	60.7	16	287	74.6	16	16.8%	18.00 [-29.12, 65.12]
Hwang 2023	246.49	79.69	41	224	76.39	41	32.8%	22.49 [-11.30, 56.28]
Park 2015	290	93.4	41	288.4	99.9	41	21.3%	1.60 [-40.26, 43.46]
**Total (95% CI)**			**115**			**115**	**100.0%**	**16.44 [-2.90, 35.77]**

Heterogeneity: Chi² = 0.64, df = 3 (P = 0.89); I² = 0%
Test for overall effect: Z = 1.67 (P = 0.10)

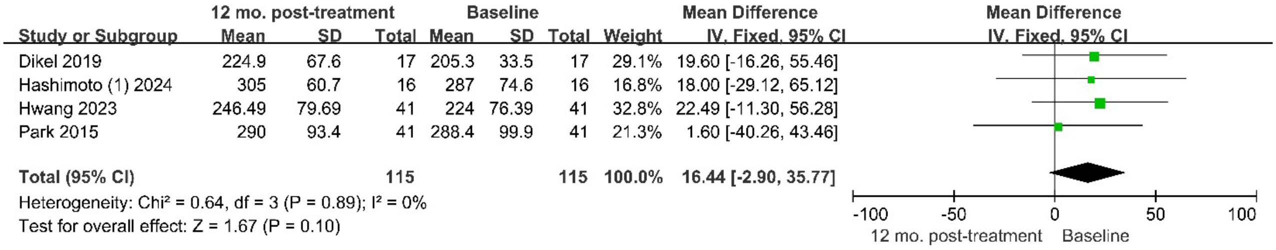

Supplementary Figure 4. Forest plots of choroidal thickness changes at different times from baseline to after treatment after deleting the literature that contributed significantly to heterogeneity.
